# Genome-wide association study of outcrossing in cytoplasmic male sterile lines of rice

**DOI:** 10.1038/s41598-017-03358-9

**Published:** 2017-06-12

**Authors:** Liang Guo, Fulin Qiu, Harish Gandhi, Suresh Kadaru, Erik Jon De Asis, Jieyun Zhuang, Fangming Xie

**Affiliations:** 1 State Key Laboratory of Rice Biology and Chinese National Center for Rice Improvement, China National Rice Research Institute, Hangzhou, 310006 China; 2 International Rice Research Institute, DAPO Box 7777, 1301 Metro Manila, Philippines; 3 Liaoning Rice Research Institute, Shenyang, 110101 China; 4 Syngenta India Ltd., Medchal Mandal, R.R. District, TS 501401 India; 5 Yuan Longping High-Tech Agriculture Co. Ltd., Changsha, 410000 China

## Abstract

Stigma exsertion and panicle enclosure of male sterile lines are two key determinants of outcrossing in hybrid rice seed production. Based on 43,394 single nucleotide polymorphism markers, 217 cytoplasmic male sterile lines were assigned into two subpopulations and a mixed-group where the linkage disequilibrium decay distances varied from 975 to 2,690 kb. Genome-wide association studies (GWAS) were performed for stigma exsertion rate (SE), panicle enclosure rate (PE) and seed-setting rate (SSR). A total of 154 significant association signals (*P* < 0.001) were identified. They were situated in 27 quantitative trait loci (QTLs), including 11 for SE, 6 for PE, and 10 for SSR. It was shown that six of the ten QTLs for SSR were tightly linked to QTLs for SE or/and PE with the expected allelic direction. These QTL clusters could be targeted to improve the outcrossing of female parents in hybrid rice breeding. Our study also indicates that GWAS-base QTL mapping can complement and enhance previous QTL information for understanding the genetic relationship between outcrossing and its related traits.

## Introduction

The availability of affordable hybrid rice seeds is crucial to the success of hybrid rice commercialization. Currently, the average yield of hybrid rice seed production in China is about 2.7 t ha^−1^. In other Asian countries, it is 1.0–1.5 t ha^−1^. Such low yields in seed production have been a major constraint to hybrid rice dissemination. Improving outcrossing, which is an important and complicated trait in determining female parent seed yield, is thus always a priority in hybrid rice breeding.

Outcrossing of rice sterile parent is a secondary and final trait resulting from flower structure and flowering characteristics. Among multi-traits affecting outcrossing, stigma exsertion and panicle enclosure are usually observed and measured as indicators of outcrossing^1, 2^ in hybrid rice breeding practice. Stigma in a rice spikelet could be exserted from the spikelet when it is flowering, and may be found outside the spikelet when the flowering is over and spikelet is closed, which increases the chance of being fertilized. Panicles of cytoplasmic male sterile (CMS) rice are partially enclosed inside the sheath and this leaves a portion of the spikelet in the panicle unavailable for pollination, with that portion of enclosed panicle being varied across genotypes.

In the past two decades, quantitative trait loci (QTLs) for stigma exsertion and panicle enclosure have been mapped by using segregating populations such as F_2_ populations^3–9^, recombinant inbred lines^10–16^, doubled haploid lines^17–19^ and backcrossing populations^14, 20–24^ These efforts have shown that these traits are of great complexity and strongly influenced by environments, resulting in slow progress in the fine-mapping of QTLs detected in primary populations.

While traditional QTL mapping using bi-parental crosses has a low resolution rate, which is usually restricted to 10–20 cM, QTL mapping with genome-wide association study (GWAS) has proven to be a promising new approach to localize QTLs in a quite precise position. GWAS is based on the availability of linkage disequilibrium (LD), known as non-random association of alleles at two or more polymorphic loci, which makes it possible to exploit the correlations between genetic markers and phenotypic variation to localize QTLs in fine-scale level^25^. In recent years, association mapping has been shown to be a useful tool in the dissection of complex trait variations in many plant species such as rice^26^, maize^27^, wheat^28^, soybean^29^ and rapeseed^30^.

GWAS-base QTL mapping has been successfully employed for a wide range of agronomically important traits in rice, including flowering time, plant height, yield traits and quality traits^31–33^. A more recent study extended the trait to stigma exsertion, showing that *GS3*, *GW5* and *GW2* play an important role in the genetic basis of stigma exsertion in rice^34^. Nevertheless, none of these studies targeted at CMS line which is an essential category of rice cultivars for hybrid rice production. Moreover, it is inconvenient to use traditional population segregation for mining QTLs controlling natural outcrossing in rice for being a self-pollinating species. Association mapping could be a potential tool for connecting genomics and phenomics in CMS rice germplasm, and fill the gap on the genetic basis of natural outcrossing. Using a collection of diverse wild abortive CMS (CMS-WA) lines, the objectives of the present GWAS study were (i) to investigate the genetic architecture of CMS lines developed at the International Rice Research Institute (IRRI) based on a 44 K single nucleotide polymorphism (SNP) genotyping array; (ii) to identify candidate genomic regions controlling outcrossing; and (iii) to characterize the genetic relationship between outcrossing and its related traits.

## Results

### Phenotype performance

The phenotypic performance of 217 rice CMS lines is presented in Table 1. A broad diversity of phenotype was observed with respect to all the traits tested. Seed-setting rate (SSR) had the highest coefficient of variation (CV), estimated as 47.9% and 38.5% in the wet season of 2013 (13WS) and dry season of 2014 (14DS), respectively. Panicle enclosure rate (PE), which was the other trait tested in two seasons, had a greater CV (19.3%) in 13WS than in 14DS (12.6%). Variances between the two seasons were highly significant (*P* < 0.0001) for the two traits, contributing 26.2% and 50.7% to the total phenotypic variance for SSR and PE, respectively (Table 2). Among the three traits for stigma exsertion rate which was measured in 14DS only, the maximum CV of 37.0% was detected for double stigma exsertion rate (DSE), followed by total stigma exsertion rate (TSE) and then single stigma exsertion rate (SSE).

**Table 1:** Phenotypic performance of five traits of 217 rice male sterile lines.

Trait	Season	Mean	SD	Range	CV (%)
Seed-setting rate (%)	13WS	12.8	6.1	2.1–32.2	47.9
	14DS	21.2	8.2	5.0–49.7	38.5
Panicle enclosure rate (%)	13WS	47.9	9.2	23.5–78.2	19.3
	14DS	33.6	4.2	17.7–42.1	12.6
Single stigma exsertion rate (%)	14DS	30.1	5.3	14.2–43.1	17.6
Double stigma exsertion rate (%)	14DS	28.4	10.5	4.9–62.9	37.0
Total stigma exsertion rate (%)	14DS	58.5	12.4	20.8–87.0	21.1

13WS = wet season of 2013; 14DS = dry season of 2014; SD = standard deviation, based on the measured values of three replications; CV = coefficient of variation, calculated as the ratio of the standard deviation to the mean value.

**Table 2:** Analysis of variance on the phenotypic performance of seed-setting rate and panicle enclosure rate.

Source of variation	Seed-setting rate		Panicle enclosure rate	
	Sum of squares	*P*	Sum of squares	*P*
Among 217 rice accessions	13926.5	1.6 × 10^−12^	13774.6	5.5 × 10^−12^
Between two seasons	6614.7	3.3 × 10^−35^	19158.3	6.4 × 10^−64^
Error	4743.1		4874.3	
Total	25284.3		37807.2	

Pearson’s product-moment correlation coefficients between the traits tested in 14DS is presented in Supplementary Table S1. The three traits evaluating stigma exsertion were positively correlated with each other, but the coefficients were high between TSE and DSE (*r* = 0.9058, *P* < 0.001), moderate between TSE and SSE (*r* = 0.5386, *P* < 0.001), and low and insignificant between DSE and SSE. This indicates that phenotypic variation of TSE among the 217 rice lines was mainly contributed by DSE, which is expected since DSE was much more variable than SSE. Accordingly, while TSE and DSE were significantly positively correlated with SSR (*P* < 0.001; *r* = 0.4171 for TSE and *r* = 0.4260 for DSE), SSE was not significantly correlated with SSR. In the meantime, SSR was significantly negatively correlated with PE (*r* = −0.2400, *P* < 0.001). The correlation of seed-setting rate with stigma exsertion rate and panicle enclosure rate was in accord with the common understanding that enhancing stigma exsertion could facilitate outcrossing whereas panicle enclosure may interfere with outcrossing.

### SNP performance and genetic diversity

The 217 CMS maintainer lines of rice, including 44 IRRI germplasm accessions and 173 breeding lines, were genotyped using a rice SNP chip. Of the 43,394 SNPs tested, 247 did not have information on physical position and 2,620 showed no polymorphism. Together with 127 SNPs having a proportion of missing data >0.2 and 15,065 SNPs with minor allele frequency <0.05, a total of 18,059 SNPs were excluded for being analyzed. The remaining 25,335 high-performing SNPs had an average of 17 kb per SNP, based on which further analysis was performed. In this rice panel, major allele frequencies varied from 0.4174 to 0.9495 with an average of 0.7230, gene diversity had an average of 0.3718 with a range from 0.0958 to 0.6472, and polymorphism information content (PIC) ranged from 0.0912 to 0.5722 with an average of 0.3052 (Supplementary Table S2). The gene diversity and PIC of the rice lines are slightly higher than those estimated using 215 diverse *indica* rice cultivars^35^. This indicates that our panel has well-captured the abundant genetic variation of the *indica* rice germplasm.

### Population structure, kinship, and LD

**Figure 1:**
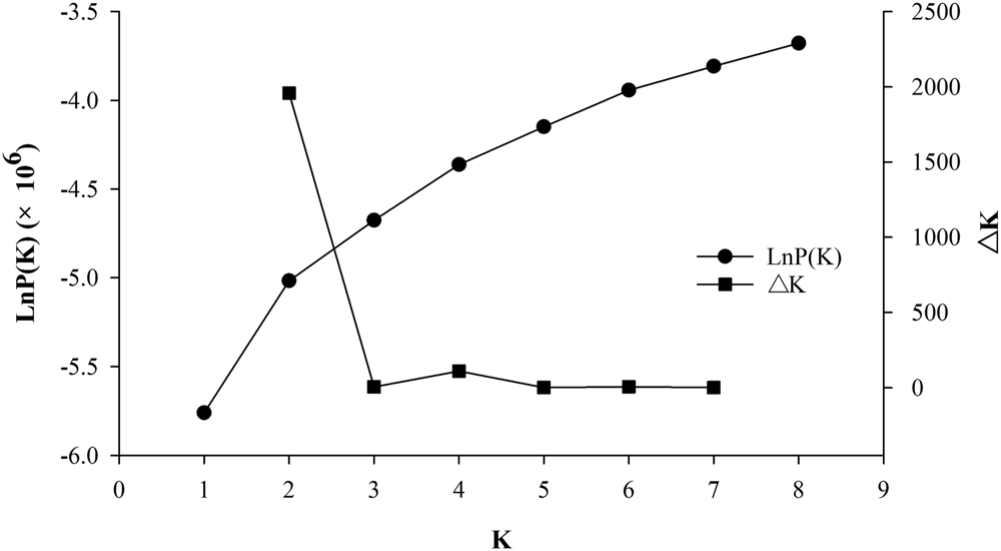
Likelihood distribution of subgroup based on model grouping method using the program STRUCTURE. Ln*P*(K) and ∆*K* plotted as a function of the assumed number of subgroups (*K*).

**Figure 2:**
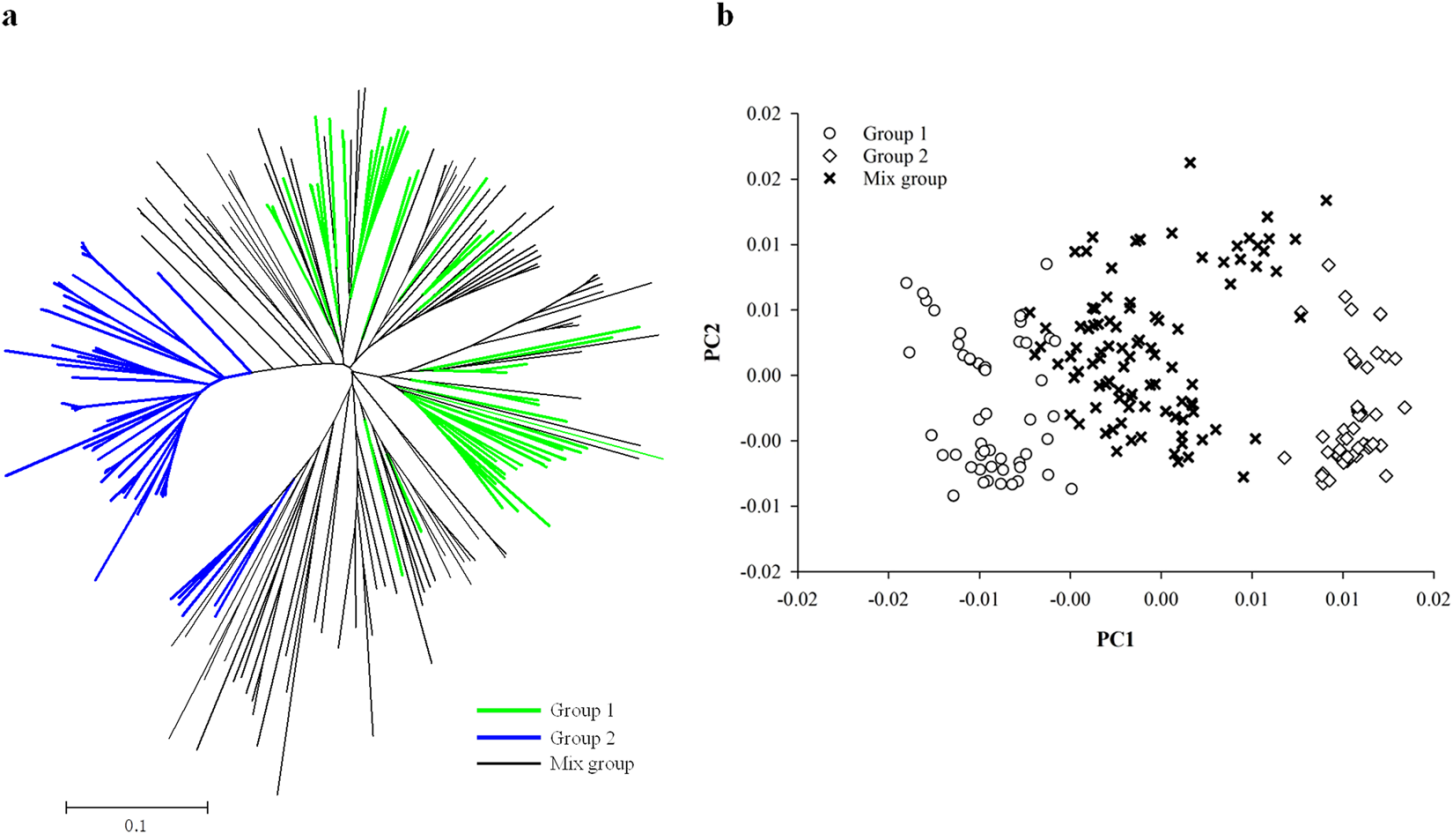
Neighbor-joining tree and PCA plot for 217 rice maintainer lines based on Cavalli-Sforza’ Chord genetic distance. (**a**) Neighbor-joining tree. (**b**) PCA plots of the first two components.

Population structure was calculated by STRUCTURE using 25,335 SNPs. The Ln*P*(K) appeared to be an increasing function of *K* as the putative *K* increased from 1 to 8, and no turning point was evident (Fig. 1). At the same time, population structure indicated by the Δ*K* function showed that *K* = 2 could be determined as the optimum *K* (Fig. 1). Using a cut-off Q-value of 0.8, 59 and 57 lines were assigned into groups 1 and 2, respectively, while the remaining 101 entries went into the mixed group (Supplementary Table S3). The numbers of entries assigned into groups 1, group 2 and the mixed group were 12, 8 and 24 for the 44 germplasm accessions, and 47, 49 and 77 for the 173 breeding lines, respectively. A large number of mixed entries no only showed a broad gene exchange between the two subgroups, but also indicated that our rice panel did not have clear population stratification. Thus, it is suitable to use a single-set data including all the 217 entries for GWAS. The separation of the two subgroups were presented as well in a neighbor-joining tree based on the Cavalli-Sforza’ Chord genetic distance, but the mixed entries were scattered over the tree (Fig. 2a). Separation between groups 1 and 2 was clearly observed in the PCA plots using the top two principal components which accounted for 18.8% and 10.1% of the genetic variation, respectively, with a large number of mixed-group entries locating between the two subgroups (Fig. 2b). The two subgroups showed significant difference (*P* < 0.001) on heading date, with the entries in group 2 delaying for an average of 5 days in 13WS and 14DS (Supplementary Fig. S1).

**Figure 3:**
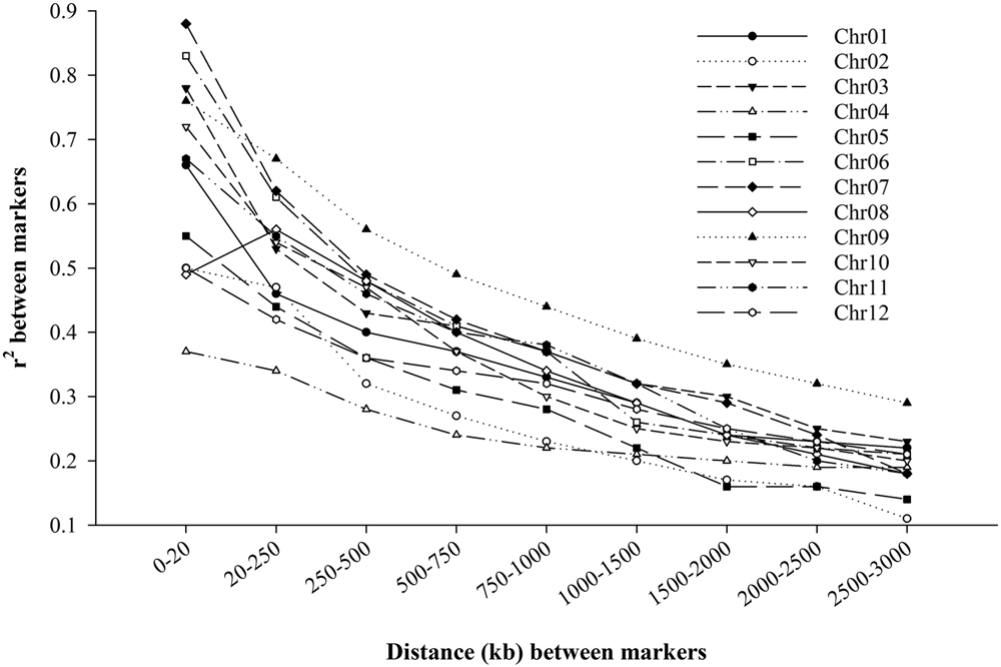
LD decay distance in each chromosome estimated from 217 rice maintainer lines.

The kinship between all pairwise samples varied from 0 to 1.6810 and averaged as 0.4556, among which 82% of the kinship coefficients had a value less than 0.6 (Supplementary Fig. S2). LD scores between markers (*r* ^*2*^) varied within and among the chromosomes (Fig. 3). The initial *r* ^*2*^ value for the 0–20 kb interval ranged from 0.37 to 0.88 and averaged as 0.64. The *r* ^*2*^ decreased with increasing genetic distance on all the 12 chromosomes. The average LD decay distance was 957 kb with a range from 577 kb on chromosome 6 to 2,690 kb on chromosome 4.

### Genome-wide association scanning

**Figure 4:**
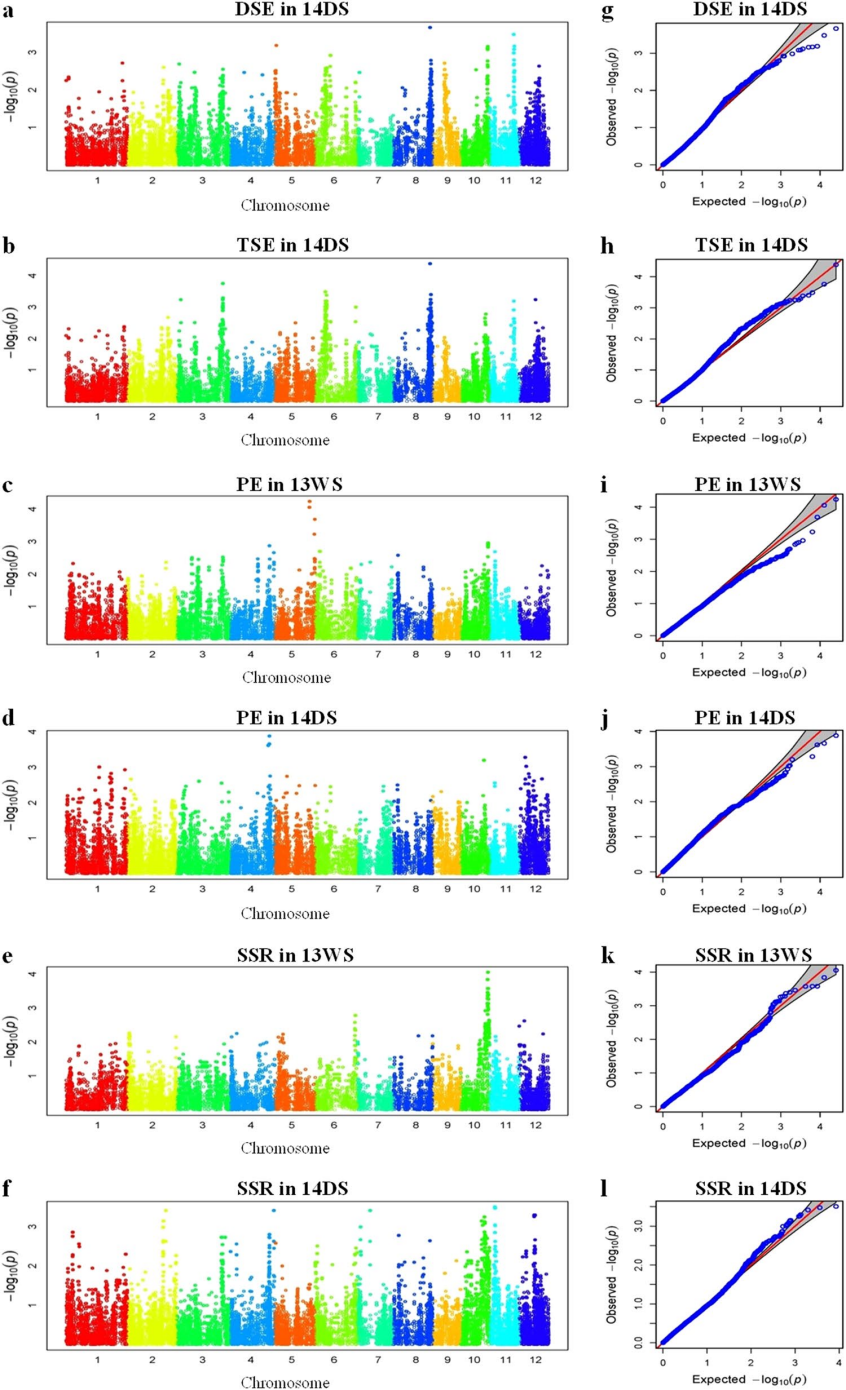
Genome-wide association study of three key traits for outcrossing in 217 cytoplasmic male sterile lines. (**a**–**f**), Manhattan plots for double stigma exsertion rate (DSE) and total stigma exsertion rate (TSE) in 14DS, and panicle enclosure rate (PE) and seed-setting rate (SSR) in wet season of 2013 (13WS) and dry season of 2014 (14DS). (**g–l**), Quantile-quantile plot. The grey areas are the 95% confidence intervals under the null hypothesis of no association between the SNP and the trait. The red line is the expected line under the null distribution. The blue points are the observed distribution.

GWAS was performed with the compressed mixed linear model controlling population structure and familial relatedness. The quantile-quantile plots (Fig. 4) showed that the distribution of observed −log10 (*P*) values matched well with the expected distribution, indicating a low rate of false positive in the detection of significant trait-marker association. A total of 154 significant associations (*P* < 0.001) were detected, including 83 for SSR, 42 for TSE, 9 for DSE, and 20 for PE (Table 3 and Fig. 4). These significant associations were distributed in all the 12 rice chromosomes except chromosome 9. To reduce the redundancy of significant associations, a shared QTL region for multiple SNPs was declared for those located within the average LD decay distance of approximately 1,000 kb, which was estimated from the 217 rice lines. As a consequence, the number of QTLs detected was reduced to 29 (Table 3).

**Table 3:** Significant association identified by GWAS.

Chr	QTL^a^	Season	Position^b^	Count^c^	−log10 (*p*)	Allele		MAF	Allelic Effect^d^	Previous study
						Major	Minor			
1	*qPE1*	14DS	25,631,546	1	3.01	A	T	0.44	−1.2	Qiao *et al*.^23^
2	*qSSR2*	14DS	30,011,758	12	3.41	C	A	0.38	−3.0	
3	*qTSE3.1*	14DS	3,496,426	1	3.24	A	G	0.36	−4.4	Qiao *et al*.^22^ and Li *et al*.^8^
	*qTSE3.2*	14DS	36,026,972	10	3.76	A	G	0.36	−4.8	Li *et al*.^19^
4	*qPE4*	14DS	30,246,596	3	3.88	A	G	0.12	2.4	
	*qSSR4*	14DS	33,677,314	3	3.41	G	A	0.38	−3.0	
5	*qDSE5*	14DS	958,555	1	3.18	A	G	0.06	−5.6	Yu *et al*.^13^
	*qPE5.1*	13WS	26,893,757	2	4.24	A	G	0.19	3.9	Qiao *et al*.^23^
	*qPE5.2*	13WS	30,785,437	2	3.69	C	A	0.08	6.2	Qiao *et al*.^23^
6	*qTSE6.1*	14DS	7,909,684	18	3.49	G	A	0.44	4.5	Shen *et al*.^12^ and Rahman *et al*.^16^
	*qTSE6.2*	14DS	31,488,536	1	3.01	G	A	0.12	−4.5	
7	*qSSR7*	14DS	9,713,206	2	3.41	A	T	0.38	−3.0	
8	*qDSE8*	14DS	28,331,096	1	3.66	G	A	0.19	−4.2	Li *et al*.^20^, Yu *et al*.^13^ and Chen *et al*.^9^
	*qTSE8.1*	14DS	28,331,096	1	4.38	G	A	0.19	−4.7	Li *et al*.^20^, Yu *et al*.^13^ and Chen *et al*.^9^
	*qTSE8.2*	14DS	28,630,523	2	3.15	C	A	0.32	3.8	Li *et al*.^20^, Yu *et al*.^13^ and Chen *et al*.^9^
	*qTSE8.3*	14DS	29,044,007	7	3.40	G	A	0.29	−3.8	Li *et al*.^20^, Yu *et al*.^13^ and Chen *et al*.^9^
	*qSSR8*	13WS	30,338,690	1	3.32	A	G	0.30	−2.0	
10	*qSSR10.1*	13WS	15,076,397	2	3.34	C	A	0.42	2.0	
	*qSSR10.2*	14DS	16,307,723	2	3.14	A	G	0.40	2.8	
	*qPE10*	14DS	17,927,625	9	3.20	A	G	0.05	−2.2	
	*qSSR10.3*	14DS	18,411,993	4	3.25	A	G	0.35	2.8	
	*qDSE10*	14DS	20,858,957	4	3.16	G	A	0.23	3.4	Uga *et al*.^10^ and Li *et al*.^15^
	*qSSR10.4*	13WS	21,100,896	40	4.05	A	G	0.17	2.9	
11	*qSSR11*	14DS	4,233,914	10	3.50	A	T	0.38	−3.1	
	*qTSE11*	14DS	18,887,723	1	3.19	G	C	0.40	3.9	
	*qDSE11*	14DS	18,887,723	3	3.47	G	C	0.40	4.0	
12	*qPE12*	14DS	4,769,088	3	3.28	G	A	0.06	−2.4	
	*qSSR12*	14DS	11,709,040	7	3.30	T	A	0.23	4.0	
	*qTSE12*	14DS	12,827,174	1	3.24	G	A	0.36	−4.4	

Chr = Chromosome number; 13WS = wet season of 2013; 14DS = dry season of 2014; MAF = minor allele frequency. ^a^QTL was designated following the nomenclature proposed by McCouch and CGSNL^51^; ^b^Position of the SNP showing the most significant association in the QTL region; ^c^Amount of significant trait-SNP association (*P* < 0.001) detected in the region; ^d^A positive value of allelic effect means that the minor allele has a higher value.

For stigma exsertion rate which was tested in 14DS only, four and nine QTLs were detected for DSE and TSE, respectively, and none was found for SSE. The strongest signal was found at the same 28,331,096 bp locus on chromosome 8 for the two traits. Major alleles of the two QTLs, *qDSE8* and *qTSE8.1*, increased DSE and TSE by 4.2% and 4.7%, respectively. The QTL with the second strongest signal for DSE, *qDSE11*, also matched a QTL for TSE, *qTSE11*, with the minor allele increasing DSE and TSE by 3.9% and 4.0%, respectively. Such consensus was in accord with the high correlation between DSE and TSE. Since DSE and TSE are two related estimates for stigma exsertion, *qDSE8*/*qTSE8.1* and *qDSE11*/*qTSE11* could each be regarded as one QTL. Thus, the total number of QTLs detected in this study was reduced to 27, and the QTL number for stigma exsertion rate reduced to 11. Two other QTLs for DSE were located on chromosomes 5 and 10, and seven other QTLs for TSE were distributed on chromosomes 3, 6, 8, and 12. The major alleles promoted stigma exsertion at *qDSE5*, *qTSE3.1*, *qTSE3.2*, *qTSE6.2*, *qTSE8.3*, and *qTSE12*, with the allelic effects ranging from 3.8% to 5.6%. The minor allele promoted stigma exsertion at *qDSE10*, *qTSE6.1*, and *qTSE8.2*, with the allelic effects ranging from 3.4% to 4.5%.

For panicle enclosure rate, two and four QTLs were identified in 13WS and 14DS, respectively. None of them were commonly detected in the two trials. The two QTLs detected in 13WS, *qPE5.1* and *qPE5.2*, were located in the regions of 26.7−26.9 and 30.6−30.8 Mb on chromosome 5, with the major alleles reducing PE by 3.9% and 6.2%, respectively. The four QTLs detected in 14DS were located on chromosomes 1, 4, 10, and 12, respectively, with the major allele at *qPE4* and minor alleles at *qPE1*, *qPE10*, and *qPE12* reducing PE by 1.2%~2.4%.

For SSR, three and seven QTLs were identified in 13WS and 14DS, with the allelic effects ranging as 2.0%~2.9% and 2.8%~4.0%, respectively. Three of the QTLs, *qSSR2*, *qSSR7*, and *qSSR11*, were independently segregated from QTLs for other traits found in this study. While no QTLs for PE and stigma exsertion rate were identified on chromosomes 2 and 7, the two QTLs for stigma exsertion rate located on chromosome 11 were 14.4-Mb apart from *qSSR11*. The major alleles of the three QTLs for SSR all had enhancing effects.

Four other QTLs for SSR, *qSSR10.1*, *qSSR10.2*, *qSSR10.3*, and *qSSR10.4*, were linked in the 14.6−21.4 Mb region on chromosome 10. The minor alleles of these QTLs all increased SSR. It was found that two QTLs for other traits, *qPE10* and *qDSE10*, were also located in this region, with the minor alleles reducing panicle enclosure rate and increasing stigma exsertion rate, respectively. Again, this was in accord with the unfavorable and favorable influence of panicle enclosure and stigma exsertion on outcrossing, respectively.

The remaining three QTLs for SSR were distributed on chromosomes 4, 8, and 12. Each of them was linked to a QTL for panicle enclosure rate or stigma exsertion rate, and the expected association was observed for two pairs of the QTLs. While the major alleles of *qSSR4* and *qSSR8* increased SSR, those of *qPE4* and *qTSE8.3* were favorable for reducing PE and increasing TSE, respectively. On the other hand, the minor alleles of *qSSR12* and *qTSE12* were favorable for increasing SSR and unfavorable for increasing TSE, respectively, which is an exemption for the consensus between SSR and related traits.

## Discussion

In this paper, the first result of GWAS for key outcrossing-related traits in rice was reported. Using the rice SNP chip consisting of 43,394 SNPs, the genetic diversity, LD pattern, population structure, and kinship of a diverse panel of 217 rice CMS-WA lines were characterized, and QTLs responsible for three key traits determining outcrossing in rice were identified.

The LD decay distance estimated in this study was found to vary greatly among different genomic regions, ranging from 577 kb to 2690 kb over the 12 chromosomes of rice. The large variation was in conformity with the results of previous studies^36–39^. It was also found that the average LD decay distance of approximately 1000 kb in our panel was much higher than the value of 100–300 kb uncovered by previous studies^31–33^. This was in agreement with the understanding that selection pressure could result in extending LD^40^. While a collection representing the entire diversity of Chinese landrace was used by Huang *et al*.^31^ and a global collection of diverse rice varieties was used by Huang *et al*.^32^ and Zhao *et al*.^33^, all the entries used in our study are in the category of CMS-WA and maintainer lines. Moreover, 173 of the total 217 lines are breeding lines selected from an on-going breeding program at IRRI.

The three key traits for outcrossing in rice were subjected to GWAS-base QTL mapping in this study, among which seed-setting rate is the direct measurement of outcrossing efficiency while stigma exsertion and panicle enclosure are critical factors determining outcrossing. A total of 27 QTLs were detected, including 10 for seed-setting rate, 11 for stigma exsertion rate, and 6 for panicle enclosure rate. Eleven of the QTLs were located in genomic regions where QTLs for the same trait was previously detected, including *qTSE3.1*, *qTSE3.2*, *qDSE5*, *qTSE6.1*, *qDSE8*/*qTSE8.1*, *qTSE8.2*, *qTSE8.3*, and *qDSE10* for stigma exsertion rate and *qPE1*, *qPE5.1*, and *qPE5.2* for panicle enclosure rate (Table 3). It is noted that the strongest signal for stigma exsertion rate, *qDSE8*/*qTSE8.1*, has been reported in multiple studies^9, 13, 20^. The remaining six QTLs for stigma exsertion rate or panicle enclosure rate, *qTSE6.2*, *qDSE11*/*qTSE11*, *qTSE12*, *qPE4*, *qPE10*, and *qPE12*, are newly detected in this study. Regarding seed-setting rate as a measurement for outcrossing, no QTL has been reported before, thus new information is provided by the detection of QTL for this trait in the present study.

As it is well known, stigma exsertion plays a vital role in promoting outcrossing, while panicle enclosure has the opposite influence. In this study, seed-setting rate was found to be positively and negatively significantly correlated with stigma exsertion rate and panicle enclosure rate, respectively. Allelic directions of individual QTLs of the QTL clusters detected in this study has provided the genetic basis for this phenomenon. Seven of the QTLs detected for seed-setting rate were closely linked to QTLs for stigma exsertion rate or/and panicle enclosure rate. In all cases except the *qSSR12*/*qTSE12* cluster, linked QTLs had the same allelic direction for seed-setting rate and stigma exsertion rate but opposite direction for seed-setting rate and panicle enclosure rate. QTL cluster of this kind is in high demand for marker-assisted breeding since several correlated traits could be selected at the same time to raise the breeding efficiency.

## Methods

### Plant materials

A total of 217 rice CMS-WA lines and their corresponding maintainer lines were used, among which 44 pairs were breeding-true lines which have been designated the accession numbers of IRRI germplasm bank, while the remaining 173 pairs were from IRRI CMS-WA breeding project which were documented by the pedigree (Supplementary Table S3). The 173 unnamed maintainers were breeding lines in the F_12_~F_18_ generations of 69 crosses and their male sterile lines are completely sterile. The maintainer lines were used for genotyping and as the pollinator in evaluating the seed-setting rate of the CMS plants, and the CMS lines were used for trait measurement.

### SNP genotyping

The 217 maintainer lines were subjected to genotyping using genomic DNA from the fresh leaves of 28-day-old plants. SNP genotyping using the rice SNP chip consisting of 43,394 SNPs was provided by Syngenta India. SNPs without physical position information and those having call frequency < 0.8 or allele frequency < 0.05 were excluded.

### Field experiments

**Figure 5:**
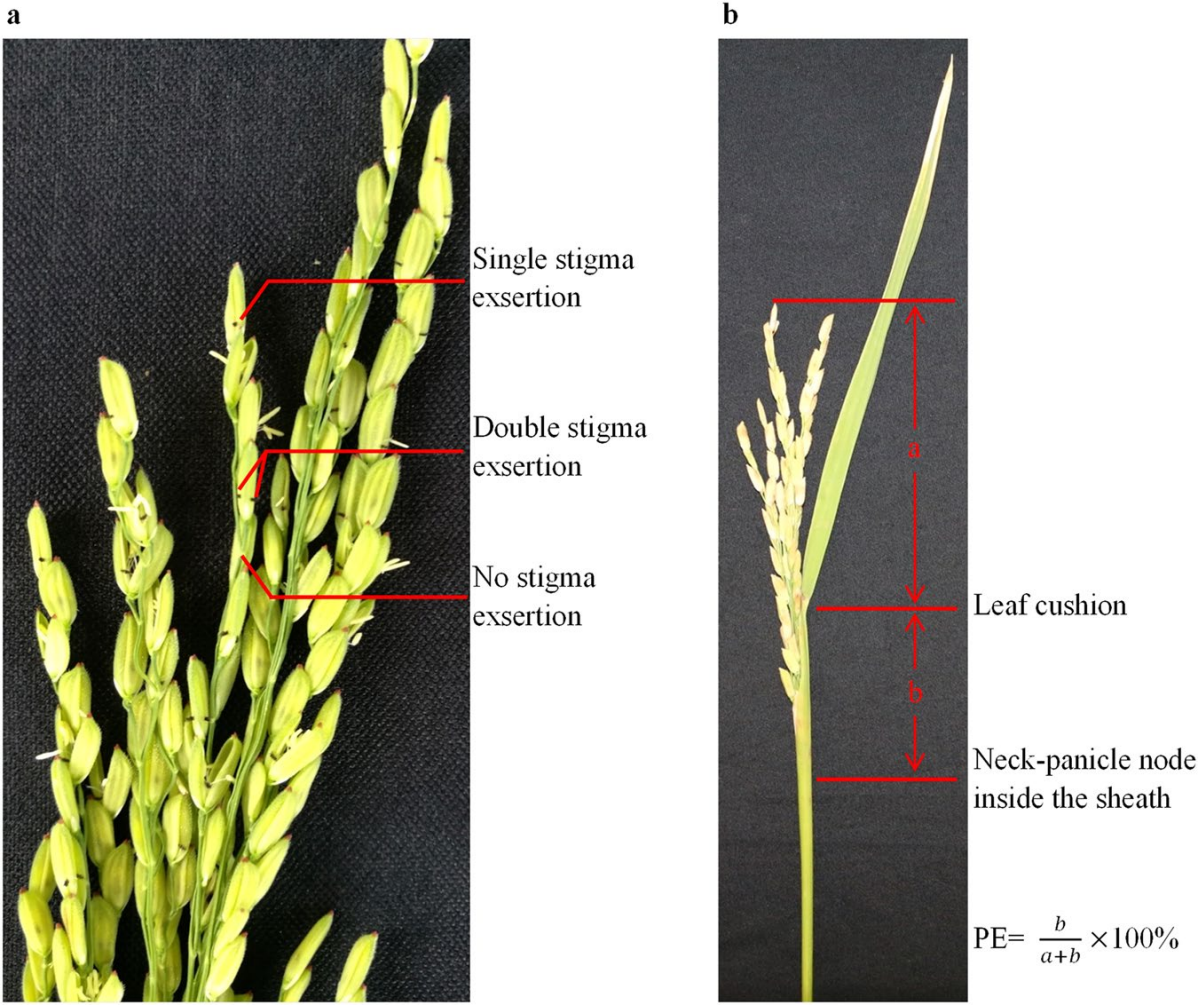
Phenotyping of stigma exsertion and panicle enclosure in this study. (**a**) stigma exsertion. (**b**) panicle enclosure.

The 217 pairs of sterile and maintainer lines were tested at the experiment station of IRRI located in Los Banos, Philippines for two seasons, namely, the wet season of 2013 (13WS) and the dry season of 2014 (14DS). The maintainer lines were sown three days later than the sterile lines. They were grown in a randomized complete block design with three replications. Twenty-one day old seedlings were transplanted using a planting density of 20 cm × 20 cm. Each pair was grown in five rows with 10 plants per row, of which the middle three rows were for sterile lines and the rest for maintainers. Field management followed local recommendations for the two different cropping seasons. Heading date was scored for individual plants and averaged for each sterile line and maintainer line in each replication. Stigma exsertion rate, panicle enclosure rate, and seed-setting rate were measured for each sterile line, with stigma exsertion rate being evaluated in 14DS only while the two other traits were determined in both seasons. For measuring the stigma exsertion rate of a sterile line, five panicles from five plants in each replication were selected on the third day after flowering. After the removal of the spikelets that were not yet flowering, spikelets with no stigma exsertion, single stigma exsertion, and double stigma exsertion (Fig. 5a) were counted, respectively. The single stigma exsertion rate (SSE), double stigma exsertion rate (DSE), and total stigma exsertion rate (TSE) were calculated as the percentage of the numbers of spikelets with single stigma exsertion, double stigma exsertion, and either single or double stigma exsertion in the total number of spikelets, respectively. For panicle enclosure rate (PE), the scoring samples were eight panicles from five plants per sterile line at maturity. PE, referring to panicle enclosed inside the sheath, was defined as the rate of the distance between the neck-panicle node and the flag leaf-cushion to the distance between the neck-panicle node to the panicle peak (Fig. 5b). Meanwhile, five healthy sterile lines in the central rows were bulk-harvested and measured for seed-setting rate (SSR).

### Statistical analysis

By using the PowerMarker version 3.25^41^, major allele frequency^42^, gene diversity^42^ and polymorphism information content (PIC)^43^ were calculated, and neighbor-joining trees was constructed based on the Cavalli-Sforza’s Chord genetic distance^44^ and viewed in MEGA version 5^45^. Population stratification was identified using STRUCTURE version 2.3.4^46^ with the parameter settings as described by Xie *et al*.^35^. The uppermost level of the assumed number of genetic groups (*K*) was determined by Δ*K* ^47^. An individual was assigned to a specific group if its genome composition derived from the group (Q-value) is above 80%; otherwise, it was assigned to the mixed group. LD was estimated using TASSEL version 5^48^ with permutations of 1,000. By using the R package GAPIT^49^, kinship was calculated, and association scan was carried out with the compressed mixed linear model^50^ where Q-value and kinship were included as the fixed and random effects, respectively. The value of *P* < 0.001 was used to declare a SNP-trait association.

### Data availability

Data for the physical positions of the 43,394 SNPs used in this study are available from the Dryad Digital Repository: http://dx.doi.org/10.5061/dryad.1tp63

## Electronic supplementary material

Supplementary figures and tables
