# Supplementary material for: Genome-wide association study of outcrossing in cytoplasmic male sterile lines of rice
Source: Sci Rep. 2017 Jun 12;7:3223. doi: 10.1038/s41598-017-03358-9 (PMC5468336; doi:10.1038/s41598-017-03358-9)
Supplement: Supplementary file 1 — Supplementary figures and tables [file 41598_2017_3358_MOESM1_ESM.doc]

**Genome-wide association study of outcrossing in cytoplasmic male sterile lines of rice**

Liang Guo^1,2^, Fulin Qiu^2,3^, Harish Gandhi^4^, Suresh Kadaru^4^, Erik Jon De Asis^2^, Jieyun Zhuang^1,*^ & Fangming Xie^2,5,*^

^1^State Key Laboratory of Rice Biology and Chinese National Center for Rice Improvement, China National Rice Research Institute, Hangzhou 310006, China

^2^International Rice Research Institute, DAPO Box 7777, 1301 Metro Manila, Philippines

^3^Liaoning Rice Research Institute, Shenyang 110101, China

^4^Syngenta India Ltd., Medchal Mandal, R.R. District, TS, 501401, India

^5^Yuan Longping High-Tech Agriculture Co. Ltd., Changsha 410000, China

^*^ Correspondence and requests should be addressed to J. Z. (email: zhuangjieyun@caas.cn) or F. X. (email: xfm@lpht.com.cn)


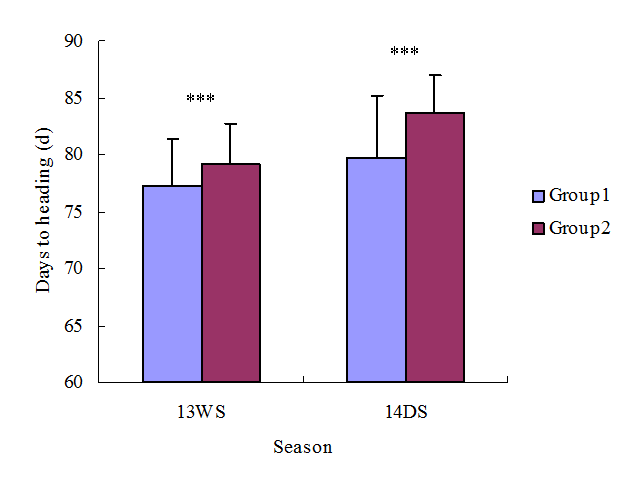


Supplementary Figure S1. Comparison of heading date between two subgroups across two seasons. Each column represents mean + s.d.. *** Significant at *P* < 0.001.

Supplementary Figure S2. Frequency distribution of pairwise kinship value between the 217 rice lines.

Supplementary Table S1. Pearson's correlation coefficients between the five traits in 14DS.

| Trait | SSR | TSE | DSE | SSE |
| --- | --- | --- | --- | --- |
| PE | -0.2400*** | -0.1136 | -0.1008 | -0.0656 |
| SSR |  | 0.4171*** | 0.4260*** | 0.1293 |
| TSE |  |  | 0.9058*** | 0.5386*** |
| DSE |  |  |  | 0.1310 |

PE = panicle enclosure rate; SSR = seed-setting rate; TSE = total stigma exsertion rate; DSE = double stigma exsertion rate; SSE = single stigma exsertion rate; *** Significant at *P* < 0.001.

Supplementary Table S2. Diversity summary statistics estimated using PowerMarker.

| Measures | Min | Max | Mean |
| --- | --- | --- | --- |
| Major allele frequency | 0.4174 | 0.9495 | 0.7230 |
| Gene diversity | 0.0958 | 0.6472 | 0.3718 |
| Polymorphism information content | 0.0912 | 0.5722 | 0.3052 |

Supplementary Table S3. Classification of 217 CMS maintainers.

| Code | Pedigree & Released accession | Cross | Group | Group membership | |
| --- | --- | --- | --- | --- | --- |
|  |  |  |  | group 1 | group 2 |
| B001 | IR85490-3-2-1-1-1-5-1-1-1-1-1-1-1-1-1-1-1 | ALAN/IR68886B-4 | 1 | 0.859 | 0.141 |
| B005 | IR85519-17-2-1-1-1-1-1-1-1-1-1-1-1-1-1 | IR68886B-1/IR78359B-7 | M | 0.566 | 0.434 |
| B007 | IR85527-23-1-1-1-1-1-1-1-1-1-1-1-1-1-1 | IR70368B-2/IR68886B-4 | 1 | 0.843 | 0.157 |
| B008 | IR85566-3-1-1-1-1-1-1-1-1-1-1-1-1-1-1 | IR80157B-11/IR78359B-7 | 1 | 1.000 | 0.000 |
| B009 | IR85566-3-1-1-2-2-1-1-1-1-1-1-1-1-1-1 | IR80157B-11/IR78359B-7 | 1 | 1.000 | 0.000 |
| B010 | IR85528-1-1-1-1-1-1-1-1-1-1-1-1-1-1-1 | IR70368B-2/IR79128B-9 | M | 0.657 | 0.343 |
| B011 | IR85521-17-3-1-1-1-3-1-1-1-1-1-1-1-1-1 | IR68886B-4/IR73321B-2 | M | 0.684 | 0.316 |
| B012 | IR85528-2-1-1-2-2-1-1-1-1-1-1-1-1-1-1 | IR70368B-2/IR79128B-9 | M | 0.736 | 0.264 |
| B013 | IR85528-5-2-1-1-2-1-1-1-1-1-1-1-1-1-1 | IR70368B-2/IR79128B-9 | M | 0.587 | 0.413 |
| B014 | IR85528-33-2-1-1-1-1-1-1-1-1-1-1-1-1-1 | IR70368B-2/IR79128B-9 | M | 0.596 | 0.404 |
| B015 | IR85552-19-1-1-1-1-1-1-1-1-1-1-1-1-1-1 | IR77819-9-1-2/IR73321B-1 | 2 | 0.000 | 1.000 |
| B016 | IR85552-37-1-1-1-1-1-1-1-1-1-1-1-1-1-1 | IR77819-9-1-2/IR73321B-1 | 2 | 0.055 | 0.945 |
| B017 | IR85552-37-1-1-1-2-1-1-1-1-1-1-1-1-1-1 | IR77819-9-1-2/IR73321B-1 | 2 | 0.055 | 0.945 |
| B018 | IR85560-18-1-2-1-1-1-1-1-1-1-1-1-1-1-1 | IR79128B-8/IR73321B-1 | M | 0.404 | 0.596 |
| B019 | IR86529-6-2-1-1-1-1-1-1-1-1-1-1-1-1-1 | IR76767B/IR81272-33-3 | M | 0.482 | 0.518 |
| B020 | IR86529-6-2-2-1-1-1-1-1-1-1-1-1-1-1-1 | IR76767B/IR81272-33-3 | M | 0.511 | 0.489 |
| B021 | IR86581-11-2-2-1-1-1-1-1-1-1-1-1-1-1 | LAGRUE/IR81272-33-3 | M | 0.711 | 0.289 |
| B022 | IR86573-7-5-1-2-1-1-1-1-1-1-1-1-1-1 | IR81278-78-3/IR79156B | 2 | 0.000 | 1.000 |
| B023 | IR86606-15-6-1-1-1-1-1-1-1-1-1-1-1-1 | MAYBELLE/IR81278-78-3 | M | 0.543 | 0.457 |
| B024 | IR86486-6-3-1-1-1-1-1-1-1-1-1-1-1-1-1 | IR69625B/IR73321B | M | 0.543 | 0.457 |
| B026 | IR86558-2-4-1-1-1-1-1-1-1-1-1-1-1-1 | IR81272-46-1/IR72789B | M | 0.663 | 0.337 |
| B027 | IR86559-5-4-1-1-1-1-1-1-1-1-1-1-1-1 | IR81272-46-1/IR73321B | M | 0.418 | 0.582 |
| B028 | IR86561-1-1-1-3-1-1-1-1-1-1-1-1-1-1 | IR81272-46-1/IR80370-C1-1-1-2-1 | M | 0.418 | 0.582 |
| B029 | IR86561-1-4-1-3-1-1-1-1-1-1-1-1-1-1 | IR81272-46-1/IR80370-C1-1-1-2-1 | M | 0.444 | 0.556 |
| B030 | IR86561-3-1-1-1-1-1-1-1-1-1-1-1-1-1 | IR81272-46-1/IR80370-C1-1-1-2-1 | M | 0.583 | 0.417 |
| B031 | IR86458-3-5-1-4-1-1-1-1-1-1-1-1-1-1 | IR58025B/IR81278-78-3 | 2 | 0.000 | 1.000 |
| B032 | IR86479-11-4-1-1-2-1-1-1-1-1-1-1-1-1 | IR68886B/IR81275-12-3 | M | 0.603 | 0.397 |
| B033 | IR86570-4-5-1-1-1-1-1-1-1-1-1-1-1-1 | IR81278-78-3/IR78376B | 2 | 0.000 | 1.000 |
| B034 | IR86571-8-5-1-1-2-1-1-1-1-1-1-1-1-1 | IR81278-78-3/IR79127B | 2 | 0.000 | 1.000 |
| B035 | IR86571-8-5-1-3-1-1-1-1-1-1-1-1-1-1 | IR81278-78-3/IR79127B | 2 | 0.000 | 1.000 |
| B036 | IR86572-3-4-1-1-1-1-1-1-1-1-1-1-1-1 | IR81278-78-3/IR79128B | 2 | 0.000 | 1.000 |
| B037 | IR86572-13-3-1-1-1-1-1-1-1-1-1-1-1-1 | IR81278-78-3/IR79128B | 2 | 0.000 | 1.000 |
| B038 | IR86573-6-1-1-1-1-1-1-1-1-1-1-1-1-1 | IR81278-78-3/IR79156B | 2 | 0.010 | 0.990 |
| B039 | IR85518-1-4-2-1-2-5-3-1-1-1-1-1-1-1-1-1-1 | IR68885B-2/IR68886B-4 | M | 0.777 | 0.223 |
| B040 | IR85565-6-3-1-1-2-2-1-1-1-1-1-1-1-1-1-1-1 | IR80157B-11/IR73321B-2 | M | 0.730 | 0.270 |
| B041 | IR86558-1-5-2-1-1-1-1-1-1-1-1-1-1-1 | IR81272-46-1/IR72789B | M | 0.765 | 0.235 |
| B042 | IR86559-9-5-1-1-1-1-1-1-1-1-1-1-1-1 | IR81272-46-1/IR73321B | 1 | 1.000 | 0.000 |
| B043 | IR86560-5-2-1-1-1-1-1-1-1-1-1-1-1-1 | IR81272-46-1/IR78376B | M | 0.633 | 0.367 |
| B044 | IR86560-9-5-1-1-1-1-1-1-1-1-1-1-1-1 | IR81272-46-1/IR78376B | 1 | 1.000 | 0.000 |
| B045 | IR86561-3-3-1-2-1-1-1-1-1-1-1-1-1-1 | IR81272-46-1/IR80370-C1-1-1-2-1 | M | 0.554 | 0.446 |
| B047 | IR86547-2-4-2-1-1-1-1-1-1-1-1-1-1-1 | IR80155B/IR68886B | 1 | 0.801 | 0.199 |
| B048 | IR86468-6-1-1-2-1-1-1-1-1-1-1-1-1-1 | IR68886B/IR71218-3-2-3 | M | 0.687 | 0.313 |
| B049 | IR86463-6-4-1-3-1-1-1-1-1-1-1-1-1-1 | IR68886B/IR75084-57-1-B-B-B | M | 0.590 | 0.410 |
| B050 | IR86472-6-1-1-3-1-1-1-1-1-1-1-1-1-1 | IR68886B/IR76767B | M | 0.543 | 0.457 |
| B051 | IR86472-6-5-2-1-1-1-1-1-1-1-1-1-1-1 | IR68886B/IR76767B | M | 0.554 | 0.446 |
| B052 | IR86475-2-2-1-3-1-1-1-1-1-1-1-1-1-1 | IR68886B/IR80155B | M | 0.671 | 0.329 |
| B053 | IR86566-1-8-2-1-1-1-1-1-1-1-1-1-1-1 | IR81278-49-2/IR81272-33-3 | M | 0.651 | 0.349 |
| B054 | IR86566-7-4-2-1-1-1-1-1-1-1-1-1-1-1 | IR81278-49-2/IR81272-33-3 | M | 0.675 | 0.325 |
| B055 | IR86456-9-1-1-1-1-1-1-1-1-1-1-1-1-1 | IR58025B/IR81272-33-3 | 1 | 0.950 | 0.050 |
| B056 | IR86458-3-4-2-1-1-1-1-1-1-1-1-1-1-1 | IR58025B/IR81278-78-3 | M | 0.597 | 0.403 |
| B057 | IR86479-8-4-1-1-1-1-1-1-1-1-1-1-1-1 | IR68886B/IR81275-12-3 | 2 | 0.000 | 1.000 |
| B058 | IR86579-2-4-1-1-1-1-1-1-1-1-1-1-1-1 | LAGRUE/IR68886B | 2 | 0.076 | 0.924 |
| B059 | IR86568-1-1-1-2-1-1-1-1-1-1-1-1-1-1 | IR81278-78-3/IR73321B | 2 | 0.075 | 0.925 |
| B060 | IR86571-11-1-4-2-1-1-1-1-1-1-1-1-1-1 | IR81278-78-3/IR79127B | 2 | 0.000 | 1.000 |
| B061 | IR86571-11-2-2-1-1-1-1-1-1-1-1-1-1-1 | IR81278-78-3/IR79127B | 2 | 0.129 | 0.871 |
| B062 | IR86572-9-4-1-1-1-1-1-1-1-1-1-1-1-1 | IR81278-78-3/IR79128B | 2 | 0.000 | 1.000 |
| B063 | IR86572-13-4-1-1-1-1-1-1-1-1-1-1-1-1 | IR81278-78-3/IR79128B | 2 | 0.000 | 1.000 |
| B064 | IR86572-13-4-1-2-1-1-1-1-1-1-1-1-1-1 | IR81278-78-3/IR79128B | M | 0.289 | 0.711 |
| B065 | IR86572-13-4-1-3-1-1-1-1-1-1-1-1-1-1 | IR81278-78-3/IR79128B | 2 | 0.000 | 1.000 |
| B066 | IR86573-1-1-1-1-1-1-1-1-1-1-1-1-1-1 | IR81278-78-3/IR79156B | 2 | 0.045 | 0.955 |
| B067 | IR86604-2-4-1-2-1-1-1-1-1-1-1-1-1-1 | MAYBELLE/IR81272-33-3 | 2 | 0.000 | 1.000 |
| B068 | IR85567-12-3-1-1-1-3-1-1-B-1-1-1-1-1-1-1-1 | IR80157B-11/IR79128B-3 | 1 | 1.000 | 0.000 |
| B069 | IR86529-6-5-2-1-1-1-1-1-1-1-1-1-1-1-1 | IR76767B/IR81272-33-3 | M | 0.469 | 0.531 |
| B070 | IR86529-10-1-1-1-1-1-1-1-1-1-1-1-1-1-1 | IR76767B/IR81272-33-3 | M | 0.320 | 0.680 |
| B073 | IR86571-8-6-2-1-1-1-1-1-1-1-1-1-1-1 | IR81278-78-3/IR79127B | 2 | 0.000 | 1.000 |
| B074 | IR86572-11-1-1-4-1-1-1-1-1-1-1-1-1 | IR81278-78-3/IR79128B | 2 | 0.000 | 1.000 |
| B075 | IR86561-1-1-1-4-1-1-1-1-1-1-1-1-1 | IR81272-46-1/IR80370-C1-1-1-2-1 | M | 0.424 | 0.576 |
| B077 | IR86559-10-1-1-1-1-1-1-1-1-1-1-1-1 | IR81272-46-1/IR73321B | 1 | 1.000 | 0.000 |
| B078 | IR86560-8-4-1-1-1-1-1-1-1-1-1-1-1 | IR81272-46-1/IR78376B | 1 | 1.000 | 0.000 |
| B079 | IR86561-1-4-1-5-1-1-1-1-1-1-1-1-1 | IR81272-46-1/IR80370-C1-1-1-2-1 | M | 0.466 | 0.534 |
| B080 | IR86458-21-9-1-3-1-1-1-1-1-1-1-1-1 | IR58025B/IR81278-78-3 | 2 | 0.000 | 1.000 |
| B081 | IR86458-3-5-1-3-1-1-1-1-1-1-1-1-1 | IR58025B/IR81278-78-3 | 2 | 0.000 | 1.000 |
| B082 | IR86479-11-7-1-1-1-1-1-1-1-1-1-1-1 | IR68886B/IR81275-12-3 | M | 0.343 | 0.657 |
| B085 | IR90983-3-1-1-1-1-1-1-1 | IR85523-6-1-1-1/IR85559-1-1-2-1-1-2 | M | 0.795 | 0.205 |
| B088 | IR90934-20-1-1-1-1-1-1-1 | IR85520-1-1-1-1-1-3/IR85491-3-3-2-1-1-1 | 1 | 1.000 | 0.000 |
| B089 | IR90937-22-1-1-1-1-1-1-1 | IR85520-1-1-1-1-1-3/IR85552-11-2-1-1 | M | 0.587 | 0.413 |
| B090 | IR90939-24-1-1-1-1-1-1-1 | IR85520-1-1-1-1-1-3/IR85560-6-2-1-1 | M | 0.555 | 0.445 |
| B091 | IR90939-33-1-1-1-1-1-1-1 | IR85520-1-1-1-1-1-3/IR85560-6-2-1-1 | M | 0.743 | 0.257 |
| B092 | IR90943-28-1-2-1-1-1-1-1 | IR85520-1-1-1-1-1-3/IR81277-60-1 | 1 | 1.000 | 0.000 |
| B093 | IR90957-10-2-1-1-1-1-1-1 | IR85491-3-3-2-1-1-1/IR85559-1-1-2-1-1-3 | 1 | 1.000 | 0.000 |
| B094 | IR90957-25-2-1-1-1-1-1-1 | IR85491-3-3-2-1-1-1/IR85559-1-1-2-1-1-3 | 1 | 1.000 | 0.000 |
| B095 | IR90958-23-2-1-1-1-1-1-1 | IR85491-3-3-2-1-1-1/IR85492-8-5-1-1-1-1 | 1 | 1.000 | 0.000 |
| B096 | IR90960-18-1-1-1-1-1-1-1 | IR85491-3-3-2-1-1-1/IR85558-2-3-1-1-2-1 | 1 | 0.902 | 0.098 |
| B097 | IR90961-2-1-1-1-1-1-1-1 | IR85491-3-3-2-1-1-1/IR85565-4-2-1-1-1-1 | 1 | 0.892 | 0.108 |
| B098 | IR90970-7-2-2-1-1-1-1-1 | IR85600-5-2-1-1/IR85521-17-2-1-1 | M | 0.715 | 0.285 |
| B099 | IR90971-11-1-1-1-1-1-1-1 | IR85600-5-2-1-1/IR81277-60-1 | M | 0.711 | 0.289 |
| B100 | IR90972-4-1-1-1-1-1-1-1 | IR85521-17-2-1-1/IR85490-3-2-1-1-1-6 | M | 0.711 | 0.289 |
| B101 | IR90974-29-1-1-1-1-1-1-1 | IR85521-17-2-1-1/IR85600-5-2-1-1 | M | 0.791 | 0.209 |
| B102 | IR90976-2-3-2-1-1-1-1-1 | IR85552-11-2-1-1/IR85559-1-1-2-1-1-3 | M | 0.462 | 0.538 |
| B103 | IR90979-1-1-1-1-1-1-1-1 | IR85570-11-1-1-1/IR85520-1-1-1-1-1-3 | 2 | 0.000 | 1.000 |
| B104 | IR90979-15-1-1-1-1-1-1-1 | IR85570-11-1-1-1/IR85520-1-1-1-1-1-3 | 2 | 0.000 | 1.000 |
| B105 | IR85567-12-3-1-1-1-3-1-1-1-1-1-1-1-1-1-1-1 | IR80157B-11/IR79128B-3 | 1 | 1.000 | 0.000 |
| B106 | IR85491-3-3-2-1-1-4-1-1-1-1-1-1-1-1-1-1-1 | ALAN/IR78359B-7 | 1 | 1.000 | 0.000 |
| B107 | IR85565-5-1-1-1-1-7-1-B-1-1-1-1-1-1-1-1-1 | IR80157B-11/IR73321B-2 | M | 0.392 | 0.608 |
| B108 | IR85552-37-1-1-1-2-1-1-1-1-1-1-1-1-1-1 | IR77819-9-1-2/IR73321B-1 | M | 0.636 | 0.364 |
| B109 | IR86606-5-2-1-2-1-1-1-1-1-1-1-1-1-1-1 | MAYBELLE/IR81278-78-3 | M | 0.495 | 0.505 |
| B110 | IR86571-4-1-1-1-1-1-1-1-1-1-1-1-1-1 | IR81278-78-3/IR79127B | 2 | 0.000 | 1.000 |
| B111 | IR86571-4-1-1-1-1-1-1-2-1-1-1-1-1-1 | IR81278-78-3/IR79127B | 2 | 0.000 | 1.000 |
| B112 | IR86571-4-1-1-1-1-1-1-2-1-1-1-1-1-1 | IR81278-78-3/IR79127B | 2 | 0.000 | 1.000 |
| B113 | IR86571-4-1-1-1-1-1-1-3-1-1-1-1-1-1 | IR81278-78-3/IR79127B | 2 | 0.000 | 1.000 |
| B114 | IR86571-4-1-1-1-1-1-1-4-1-1-1-1-1-1 | IR81278-78-3/IR79127B | 2 | 0.000 | 1.000 |
| B115 | IR86571-4-1-1-1-1-1-1-5-1-1-1-1-1-1 | IR81278-78-3/IR79127B | 2 | 0.000 | 1.000 |
| B116 | IR86572-9-3-1-1-1-1-1-1-1-1-1-1-1-1 | IR81278-78-3/IR79128B | 2 | 0.000 | 1.000 |
| B118 | IR90964-15-2-1-1-1-1-1-1 | IR85491-3-3-2-1-1-1/IR85527-12-1-1-1 | 1 | 0.864 | 0.136 |
| B119 | IR90934-13-1-1-1-1-1-1-1 | IR85520-1-1-1-1-1-3/IR85491-3-3-2-1-1-1 | 1 | 1.000 | 0.000 |
| B120 | IR90937-17-2-1-1-1-1-1-1 | IR85520-1-1-1-1-1-3/IR85552-11-2-1-1 | M | 0.481 | 0.519 |
| B121 | IR90943-24-1-1-1-1-1-1-1 | IR85520-1-1-1-1-1-3/IR81277-60-1 | 1 | 1.000 | 0.000 |
| B122 | IR90943-24-1-2-1-1-1-1-1 | IR85520-1-1-1-1-1-3/IR81277-60-1 | 1 | 1.000 | 0.000 |
| B123 | IR90955-2-1-1-1-1-1-1-1 | IR85490-3-2-1-1-1-6/IR85521-17-2-1-1 | M | 0.666 | 0.334 |
| B124 | IR90957-23-1-1-1-1-1-1-1 | IR85491-3-3-2-1-1-1/IR85559-1-1-2-1-1-3 | 1 | 1.000 | 0.000 |
| B125 | IR90958-1-1-1-1-1-1-1-1 | IR85491-3-3-2-1-1-1/IR85492-8-5-1-1-1-1 | 1 | 0.949 | 0.051 |
| B126 | IR90958-6-1-1-1-1-1-1-1 | IR85491-3-3-2-1-1-1/IR85492-8-5-1-1-1-1 | 1 | 0.832 | 0.168 |
| B127 | IR90958-12-1-1-1-1-1-1-1 | IR85491-3-3-2-1-1-1/IR85492-8-5-1-1-1-1 | 1 | 1.000 | 0.000 |
| B128 | IR90959-3-2-1-1-1-1-1-1 | IR85491-3-3-2-1-1-1/IR85518-1-7-1-1-1-1 | 1 | 1.000 | 0.000 |
| B129 | IR90959-20-1-1-1-1-1-1-1 | IR85491-3-3-2-1-1-1/IR85518-1-7-1-1-1-1 | 1 | 1.000 | 0.000 |
| B130 | IR90959-29-1-1-1-1-1-1-1 | IR85491-3-3-2-1-1-1/IR85518-1-7-1-1-1-1 | 1 | 1.000 | 0.000 |
| B131 | IR90964-9-1-5-1-1-1-1-1 | IR85491-3-3-2-1-1-1/IR85527-12-1-1-1 | 1 | 1.000 | 0.000 |
| B132 | IR90964-9-1-6-1-1-1-1-1 | IR85491-3-3-2-1-1-1/IR85527-12-1-1-1 | 1 | 0.966 | 0.034 |
| B133 | IR90964-9-1-6-1-1-1-1-1 | IR85491-3-3-2-1-1-1/IR85527-12-1-1-1 | 1 | 0.962 | 0.038 |
| B134 | IR90966-19-1-1-1-1-1-1-1 | IR85491-3-3-2-1-1-1/IR85521-17-2-1-1 | 1 | 0.929 | 0.071 |
| B135 | IR90970-12-3-2-1-1-1-1-1 | IR85600-5-2-1-1/IR85521-17-2-1-1 | M | 0.799 | 0.201 |
| B136 | IR90970-12-3-3-1-1-1-1-1 | IR85600-5-2-1-1/IR85521-17-2-1-1 | M | 0.739 | 0.261 |
| B137 | IR90972-4-1-2-1-1-1-1-1 | IR85521-17-2-1-1/IR85490-3-2-1-1-1-6 | M | 0.712 | 0.288 |
| B138 | IR90974-10-1-1-1-1-1-1-1 | IR85521-17-2-1-1/IR85600-5-2-1-1 | M | 0.641 | 0.359 |
| B139 | IR90974-19-1-1-1-1-1-1-1 | IR85521-17-2-1-1/IR85600-5-2-1-1 | 1 | 0.914 | 0.086 |
| B140 | IR90974-19-1-1-1-1-1-1-1 | IR85521-17-2-1-1/IR85600-5-2-1-1 | 1 | 0.872 | 0.128 |
| B141 | IR90979-19-1-1-1-1-1-1-1 | IR85570-11-1-1-1/IR85520-1-1-1-1-1-3 | 2 | 0.000 | 1.000 |
| B142 | IR90974-19-1-2-1-1-1-1-1 | IR85521-17-2-1-1/IR85600-5-2-1-1 | M | 0.759 | 0.241 |
| B143 | IR90980-5-1-1-1-1-1-1-1 | IR85570-11-1-1-1/IR85559-1-1-2-1-1-3 | 2 | 0.000 | 1.000 |
| B144 | IR85518-1-4-2-1-2-5-3-1-1-1-1-1-1-1-1-1-1 | IR68885B-2/IR68886B-4 | M | 0.777 | 0.223 |
| B145 | IR85599-9-1-1-1-1-3-1-1-1-1-1-1-1-1-1 | JACKSON/IR68886B-4*2 | M | 0.646 | 0.354 |
| B146 | IR85519-42-2-1-1-1-1-1-1-1-1-1-1-1-1-1 | IR68886B-1/IR78359B-7 | M | 0.710 | 0.290 |
| B147 | IR85571-6-1-1-1-1-1-1-1-1-1-1-1-1-1-1 | IR80370-C1-1-1-2-1/IR78359B-21 | M | 0.462 | 0.538 |
| B148 | IR86570-4-3-1-2-1-1-1-1-1-1-1-1-1-1 | IR81278-78-3/IR78376B | 2 | 0.000 | 1.000 |
| B149 | IR86571-8-6-1-1-1-1-1-1-1-1-1-1-1-1 | IR81278-78-3/IR79127B | 2 | 0.000 | 1.000 |
| B150 | IR86606-5-2-3-1-1-1-1-1-1-1-1-1-1-1 | MAYBELLE/IR81278-78-3 | 2 | 0.102 | 0.898 |
| B151 | IR86458-19-1-1-1-1-1-1-1-1-1-1-1-1-1 | IR58025B/IR81278-78-3 | 2 | 0.000 | 1.000 |
| B152 | IR86479-11-7-1-5-1-1-1-1-1-1-1-1-1-1 | IR68886B/IR81275-12-3 | M | 0.499 | 0.501 |
| B153 | IR86570-4-2-1-3-1-1-1-1-1-1-1-1-1-1 | IR81278-78-3/IR78376B | 2 | 0.000 | 1.000 |
| B154 | IR86572-9-3-1-1-1-1-1-1-1-1-1-1-1-1 | IR81278-78-3/IR79128B | 2 | 0.000 | 1.000 |
| B155 | IR86558-6-3-1-1-1-1-1-1-1-1-1-1-1-1 | IR81272-46-1/IR72789B | 1 | 1.000 | 0.000 |
| B156 | IR86561-3-3-1-1-2-1-1-1-1-1-1-1-1-1 | IR81272-46-1/IR80370-C1-1-1-2-1 | M | 0.535 | 0.465 |
| B157 | IR86561-4-2-1-1-1-1-1-1-1-1-1-1-1-1 | IR81272-46-1/IR80370-C1-1-1-2-1 | 1 | 0.832 | 0.168 |
| B158 | IR86547-1-1-1-1-1-1-1-1-1-1-1-1-1-1 | IR80155B/IR68886B | 1 | 0.870 | 0.130 |
| B159 | IR86551-1-1-1-1-1-1-1-1-1-1-1-1-1-1 | IR80155B/IR79156B | M | 0.681 | 0.319 |
| B160 | IR86465-3-2-1-1-1-1-1-1-1-1-1-1-1-1 | IR68886B/IR58025B | M | 0.450 | 0.550 |
| B161 | IR86463-6-4-1-2-2-1-1-1-1-1-1-1-1-1 | IR68886B/IR75084-57-1-B-B-B | M | 0.620 | 0.380 |
| B162 | IR86458-2-5-1-1-1-1-1-1-1-1-1-1-1-1 | IR58025B/IR81278-78-3 | 2 | 0.000 | 1.000 |
| B163 | IR86509-11-1-1-1-1-1-1-1-1-1-1-1-1-1 | IR72789B/IR81278-78-3 | 2 | 0.064 | 0.936 |
| B164 | IR86571-8-5-1-1-1-1-1-1-1-1-1-1-1-1 | IR81278-78-3/IR79127B | 2 | 0.000 | 1.000 |
| B165 | IR86606-5-2-1-1-1-1-1-1-1-1-1-1-1-1 | MAYBELLE/IR81278-78-3 | M | 0.462 | 0.538 |
| B166 | IR86561-1-1-1-1-1-1-1-1-1-1-1-1-1 | IR81272-46-1/IR80370-C1-1-1-2-1 | M | 0.424 | 0.576 |
| B167 | IR86456-8-1-2-1-1-1-1-1-1-1-1-1-1 | IR58025B/IR81272-33-3 | M | 0.405 | 0.595 |
| B168 | IR86570-3-4-1-1-1-1-1-1-1-1-1-1-1 | IR81278-78-3/IR78376B | 2 | 0.000 | 1.000 |
| B169 | IR90981-10-1-1-1-1-1-1-1 | IR85554-13-1-1-1/IR85559-1-1-2-1-1-3 | M | 0.575 | 0.425 |
| B170 | IR90938-4-1-1-1-1-1-1-1 | IR85520-1-1-1-1-1-3/IR85523-6-1-1-1 | M | 0.774 | 0.226 |
| B171 | IR90939-3-2-1-1-1-1-1-1 | IR85520-1-1-1-1-1-3/IR85560-6-2-1-1 | M | 0.637 | 0.363 |
| B172 | IR90939-32-1-1-1-1-1-1-1 | IR85520-1-1-1-1-1-3/IR85560-6-2-1-1 | M | 0.619 | 0.381 |
| B173 | IR90942-9-1-1-1-1-1-1-1 | IR85520-1-1-1-1-1-3/IR85511-2-2-2-1 | 1 | 0.841 | 0.159 |
| B174 | IR90977-22-1-2-1-1-1-1-1 | IR85569-15-1-1-1/IR85520-1-1-1-1-1-3 | M | 0.782 | 0.218 |
| B175 | IR90980-5-1-2-1-1-1-1-1 | IR85570-11-1-1-1/IR85559-1-1-2-1-1-3 | 2 | 0.000 | 1.000 |
| B176 | IR85520-1-3-1-1-2-1-1-B-1-1-1-1-1-1-1-1-1 | IR68886B-4/IR72790B-2 | M | 0.790 | 0.210 |
| B177 | IR85559-1-1-1-1-1-3-1-B-1-1-1-1-1-1-1-1-1 | IR79128B-3/IR68886B-4 | 1 | 1.000 | 0.000 |
| B178 | IR85520-3-2-1-1-1-3-1-B-1-1-1-1-1-1-1-1-1 | IR68886B-4/IR72790B-2 | 1 | 1.000 | 0.000 |
| B179 | IR90936-13-2-1-1-1-1-1-1 | IR85520-1-1-1-1-1-3/IR85550-12-1-1-1 | M | 0.644 | 0.356 |
| B180 | IR90943-11-1-1-1-1-1-1-1 | IR85520-1-1-1-1-1-3/IR81277-60-1 | 1 | 1.000 | 0.000 |
| B181 | IR90957-1-2-1-1-1-1-1-1 | IR85491-3-3-2-1-1-1/IR85559-1-1-2-1-1-3 | 1 | 1.000 | 0.000 |
| B182 | IR90957-33-1-1-1-1-1-1-1 | IR85491-3-3-2-1-1-1/IR85559-1-1-2-1-1-3 | 1 | 1.000 | 0.000 |
| B183 | IR90966-19-1-1-1-1-1-1-1 | IR85491-3-3-2-1-1-1/IR85521-17-2-1-1 | 1 | 0.931 | 0.069 |
| B184 | IR90970-8-3-1-1-1-1-1-1 | IR85600-5-2-1-1/IR85521-17-2-1-1 | M | 0.786 | 0.214 |
| B185 | IR90974-8-2-1-1-1-1-1-1 | IR85521-17-2-1-1/IR85600-5-2-1-1 | M | 0.675 | 0.325 |
| B186 | IR90979-6-1-1-1-1-1-1-1 | IR85570-11-1-1-1/IR85520-1-1-1-1-1-3 | 2 | 0.000 | 1.000 |
| B187 | IR90980-12-1-1-1-1-1-1-1 | IR85570-11-1-1-1/IR85559-1-1-2-1-1-3 | 2 | 0.000 | 1.000 |
| B188 | IR102560B |  | M | 0.361 | 0.639 |
| B189 | IR102562B |  | M | 0.620 | 0.380 |
| B190 | IR102563B |  | M | 0.681 | 0.319 |
| B191 | IR102564B |  | 1 | 1.000 | 0.000 |
| B192 | IR102565B |  | M | 0.617 | 0.383 |
| B193 | IR102566B |  | 1 | 0.964 | 0.036 |
| B194 | IR102567B |  | 1 | 0.924 | 0.076 |
| B195 | IR102568B |  | 1 | 0.999 | 0.001 |
| B196 | IR102569B |  | 1 | 0.835 | 0.165 |
| B197 | IR102570B |  | M | 0.790 | 0.210 |
| B198 | IR102571B |  | M | 0.605 | 0.395 |
| B199 | IR102572B |  | M | 0.698 | 0.302 |
| B200 | IR102573B |  | M | 0.241 | 0.759 |
| B201 | IR102757B |  | M | 0.360 | 0.640 |
| B202 | IR102758B |  | 2 | 0.001 | 0.999 |
| B203 | IR102759B |  | 2 | 0.143 | 0.857 |
| B204 | IR102760B |  | M | 0.605 | 0.395 |
| B205 | IR102761B |  | 1 | 0.907 | 0.093 |
| B206 | IR58025B |  | 2 | 0.000 | 1.000 |
| B207 | IR62829B |  | M | 0.374 | 0.626 |
| B208 | IR68886B |  | M | 0.361 | 0.639 |
| B209 | IR68888B |  | M | 0.304 | 0.696 |
| B210 | IR68897B |  | M | 0.208 | 0.792 |
| B211 | IR69625B |  | M | 0.497 | 0.503 |
| B212 | IR70369B |  | M | 0.489 | 0.511 |
| B213 | IR73328B |  | M | 0.366 | 0.634 |
| B214 | IR73793B |  | M | 0.335 | 0.665 |
| B215 | IR75596B |  | M | 0.352 | 0.648 |
| B216 | IR75606B |  | M | 0.269 | 0.731 |
| B217 | IR78369B |  | M | 0.346 | 0.654 |
| B218 | IR79125B |  | 2 | 0.013 | 0.987 |
| B219 | IR79156B |  | 2 | 0.082 | 0.918 |
| B220 | IR80151B |  | 2 | 0.066 | 0.934 |
| B222 | IR80559B |  | 2 | 0.001 | 0.999 |
| B223 | IR80561B |  | M | 0.337 | 0.663 |
| B224 | IR80564B |  | 2 | 0.182 | 0.818 |
| B226 | IR93559B |  | M | 0.792 | 0.208 |
| B227 | IR93560B |  | 1 | 1.000 | 0.000 |
| B228 | IR93561B |  | 1 | 1.000 | 0.000 |
| B229 | IR93562B |  | 1 | 0.867 | 0.133 |
| B230 | IR93563B |  | 1 | 1.000 | 0.000 |
| B231 | IR99790B |  | 1 | 1.000 | 0.000 |
| B232 | IR99791B |  | 1 | 0.914 | 0.086 |
| B233 | IR99792B |  | M | 0.696 | 0.304 |

Subgroup was designed by STRUCTURE. 1 = group 1; 2 = group 2; M = mixed group.
